# Advantages and Disadvantages of Bone Protective Agents in Metastatic Prostate Cancer: Lessons Learned

**DOI:** 10.3390/dj4030028

**Published:** 2016-08-19

**Authors:** Christian Thomas, Georg Bartsch, Christian Walter, Hendrik Borgmann, Maximilian Peter Brandt, Thomas Höfner, Axel Haferkamp, Igor Tsaur

**Affiliations:** 1 Department of Urology, University of Mainz, Langenbeckstraße 1, 55131 Mainz, Germany; Georg.Bartsch@unimedizin-mainz.de (G.B.); hborgmann@prostatecentre.com (H.B.); maximilian.brandt@unimedizin-mainz.de (M.P.B.); thomas.hoefner@unimedizin-mainz.de (T.H.); axel.haferkamp@unimedizin-mainz.de (A.H.); igor.tsaur@unimedizin-mainz.de (I.T.); 2 Department of Maxillofacial Surgery, University of Mainz, 55131 Mainz, Germany; walter@mainz-mkg.de

**Keywords:** zoledronic acid, denosumab, prostate cancer, osteonecrosis

## Abstract

Nine out of ten metastatic prostate cancer (PCa) patients will develop osseous metastases. Of these, every second will suffer from skeletal-related events (SRE). SRE are associated with an increased risk for death, which is markedly increased in the presence of pathological fracture. Moreover, health insurance costs nearly double in the presence of SRE. Zoledronic acid and denosumab are both approved drugs for the prevention or delay of SRE in castration-resistant prostate cancer (CRPC) patients with osseous metastases. However, long-term treatment with one of these two drugs is associated with the development of medication-related osteonecrosis of the jaw (MRONJ). Routine inspections of the oral cavity before and during treatment are mandatory in these patients. Regarding imaging techniques, bone scintigraphy seems to be a promising tool to detect early stage MRONJ. Zoledronic acid does not reduce the incidence of SRE in hormone-sensitive PCa. First data shows 3-monthly application of zoledronic acid to be equi-effective to monthly application.

## 1. Introduction

Prostate cancer (PCa) is the most frequently diagnosed cancer in men and the second leading cause of cancer death in Western countries. While less than 5% of PCa patients have clinical evidence of metastatic disease at initial diagnosis [1], approximately one-third develop PCa relapse after initial curative treatment [2]. For hormone-sensitive metastatic PCa, androgen deprivation therapy is the gold standard. However, the majority of patients will develop castration-resistant disease (CRPC) within 2–3 years [3,4]. Today, life expectancy at the CRPC state is up to 3 years [5]. Due to the high frequency of bone metastases at this stage, bone protective agents play an integral part in the prevention of skeletal-related events (SRE). Used as indicated, zoledronic acid and denosumab significantly reduce the incidence of SRE, and therefore improve the quality of life in this patient cohort.

However, several aspects regarding application, and further strategies in the case of side effects have not been fully elucidated until today. In this review, we report about the incidence of SRE in prostate cancer (PCa) patients with osseous metastases, and its economic burden. Moreover, we critically discuss the indication and application form of zoledronic acid and denosumab in PCa patients and search the current literature for further treatment approaches in the presence of medication-related osteonecrosis of the jaw (MRONJ).

## 2. Skeletal-Related Events in Metastatic Prostate Cancer: A Frequent Phenomenon and Economic Burden

PCa is the most common solid cancer in males in Western countries. In 2016, an estimated 180,890 men will be diagnosed, and 26,120 will die of the disease in the United States of America [6]. Although more than 95% of PCa are initially diagnosed in a clinically localized state, approximately 30% will progress despite initial curative treatment [1,2]. In metastatic state, up to 90% of patients will develop osseous metastases [7,8,9,10]. Of these, 40%–50% will suffer from at least one skeletal-related event (SRE). Approximately 22% will experience ≥2 SREs [11,12,13]. SREs, such as bone pain requiring palliative radiation, pathologic fractures, spinal cord compression, and the need for orthopaedic surgery, are a serious complication of osseous metastases.

SRE have a significant impact on survival in PCa patients, increasing the risk of death by 2.29-fold compared to patients without SRE [14]. In the presence of a pathological fracture, the risk of death increases by 2.77-fold.

SRE are not only associated with poor prognosis, but also with an economic burden. While in the US, costs increase by $12,780/person-year in the case of bone metastases, this amount nearly doubles in the presence of SRE [11].

## 3. Current Treatment Options for Preventing or Delaying SRE

So far, two drugs have been approved for preventing or delaying SRE in patients with metastatic castration-resistant prostate cancer (mCRPC): The bisphosphonate zoledronic acid and the receptor activator of NF-κB (RANK)-ligand inhibitor denosumab. Bisphosphonates have a long history in medicine for the prevention of bone density loss and reducing the risk of SRE. In PCa, only zoledronic acid showed superiority in the prevention of SRE compared to placebo. In a large phase III registration trial, 4 mg zoledronic acid administered i.v. every 3 weeks significantly reduced the incidence of SRE compared to placebo in mCRPC patients (44.2% vs. 33.2%; *p* = 0.021) [12]. Moreover, median time to first SRE was significantly delayed in the treatment group compared to placebo (at least 420 days vs. 321 days; *p* = 0.001). Regarding quality of life, pain and analgesic scores were higher in the placebo group compared to zoledronic acid. Interestingly, there was a strong trend towards prolonged survival in patients receiving zoledronic acid compared to placebo (546 days vs. 464 days; *p* = 0.091). Based on these results, 4 mg zoledronic acid was approved for SRE prevention in mCRPC patients with osseous metastases by the Federal Drug Administration (FDA) and the European Medicines Agency (EMA) in 2002.

Receptor Activator of NF-κB Ligand (RANKL) plays a pivotal role in bone remodelling. In detail, RANKL drives osteoclast formation, function, and survival. Denosumab is a monoclonal antibody targeting RANKL. Showing promising results in the reduction of SRE in patients with solid tumours, denosumab was tested in a large (*n* = 1901) phase III registration trial in PCa [15]. mCRPC patients with osseous metastases were prospectively randomized to denosumab 120 mg s.c. monthly or zoledronic acid at 4 mg i.v. monthly. Primary endpoint was time to first SRE. Denosumab significantly delayed time to first SRE compared to zoledronic acid (20.7 months vs. 17.1 months; *p* = 0.0002 for non-inferiority and *p* = 0.008 for superiority). First on-study SRE were 41% in the zoledronic acid arm and 36% in the denosumab arm. No clinically relevant differences were found in overall survival, disease progression, or serum PSA response. In 2011, denosumab was approved by the EMA for the prevention of SRE in patients with bone metastases from solid tumours.

## 4. Zoledronic Acid and Denosumab: Two Approved Bone Protective Agents Responsible for Medication-Related Osteonecrosis of the Jaw (MRONJ)

When zoledronic acid was approved in 2002, hypocalcaemia and renal deterioration in patients with pre-existing renal insufficiency were the only severe side effects of this drug known so far. It took one year after approval to present first evidence that zoledronic acid exposure is linked to osteonecrosis of the jaw [16]. Subsequently, several case series were reported about osteonecrosis of the jaw during bisphosphonate exposure. In the literature, MRONJ in PCa patients varies from 1.0%–18.6% [15,17,18,19,20]. Interestingly, the presence of MRONJ is related to the duration of zoledronic acid exposure. While MRONJ incidence was 1.0% in mCRPC patients receiving zoledronic acid for a median of 11.2 months, we previously reported an incidence of 18.6% in patients receiving zoledronic acid at least 14 times (median treatment duration 21.4 months) [15,17].

In daily clinical practice, a MRONJ-risk of 5% in mCRPC-patients receiving i.v. bisphosphonates seems to be realistic [21].

Denosumab is also linked to an increased incidence of MRONJ. In the denosumab-registration trial in mCRPC patients, MRONJ-incidence was 2% in the denosumab-arm (median treatment duration 12.2 months) [15]. In another study with the primary objective of delaying the development of osseous metastases in PCa-patients, MRONJ-incidence increased to 5% when receiving denosumab for at least 24 months [22].

Even incomparable to the economic burden of SRE, MRONJ treatment is associated with relevant costs. Najm and colleagues retrospectively evaluated the costs of MRONJ-treatment in 92 patients. The median cost of a case of MRONJ was $ 1,667 (interquartile range from $976–$3,350) [23].

## 5. Role of Imaging for Early Detection of Medication-Related Osteonecrosis of the Jaw

The majority of MRONJ cases are detected when the patient presents clinical symptoms. Conservative treatment with antibiotics and pain medication is an option for patients with low-stage MRONJ. However, if infection and erythema in the area of the exposed and necrotic bone are present, additional surgical debridement or resection is inevitable in the majority of patients [24]. So far, no useful clinical biomarker for the detection of MRONJ at the early stage has been described in the literature [25]. Magnetic resonance imaging (MRI) and computed tomography (CT) can be used to estimate the extent of MRONJ. Both imaging techniques have shown superiority compared to conventional panoramic radiographs [26]. However, literature is scarce regarding the role of CT and MRI in the detection of early stage MRONJ. This can be explained by the fact that CT or MRI scans of the head are not routinely performed for tumour staging/progression assessment in metastatic PCa.

Bone scintigraphy is a standard whole-body imaging technique in PCa used to assess osseous tumour burden. Routinely performed for tumour staging, bone scintigraphy might serve as a diagnostic tool for the coincidental detection of asymptomatic early stage MRONJ. In a small study of 35 MRONJ-patients, O’Ryan and colleagues have shown that bone scintigraphy had a positive tracer uptake in 67.5% of patients [27]. In another study, the detection rate of clinically symptomatic MRONJ was even higher, at 93.8% [28]. In a recently published study, we demonstrated for the first time that bone scintigraphy has the potential to detect clinically-asymptomatic MRONJ [25]. Retrospectively evaluating the presence of positive tracer uptake in the jaw in bone scans of metastatic PCa patients under bisphosphonate treatment, bone scintigraphy had an acceptable sensitivity of 67% and good specificity of 79% to detect clinically-asymptomatic MRONJ. When patients had pathologic tracer uptake of the maxilla or mandible in bone scan, the relative risk of developing MRONJ in the further clinical course was increased 7.6-fold.

In conclusion, imaging of only the head for MRONJ screening currently has no indication. However, for daily clinical routine of metastatic PCa patients receiving bone protective agents, bone scintigraphy should not only be used to assess tumour stage and treatment response, but also to check for pathological tracer uptake in the jaws.

## 6. Medication-Related Osteonecrosis of the Jaw: Stop or Proceed Treatment with Bone Protective Agents?

Unfortunately, the literature is scarce to answer the question if treatment with bone-protective agents should be stopped or proceeded in the presence of MRONJ. Watters and colleagues performed a long-term follow up of 109 patients with bisphosphonate-related osteonecrosis of the jaw [29]. Of these, 52 (48%) stopped i.v. bisphosphonate treatment. Interestingly, there was no statistical difference in the clinical course of osteonecrotic lesions in patients that discontinued bisphosphonate treatment. Rogers and colleagues started a survey of consultant members of the British Association of Oral and Maxillofacial Surgeons regarding bisphosphonate-induced osteonecrosis of the jaws [30]. Of 161 responding consultants, 39% would stop i.v. bisphosphonate medication in the presence of MRONJ. Taken together, it is unclear if bisphosphonates should be stopped in the presence of MRONJ. More data reporting about follow-up in MRONJ patients and molecular insights in bone remodelling after discontinuation of bisphosphonate treatment are needed to answer this question.

## 7. When to Start Treatment with Bone-Protective Agents: In Hormone-Naive State or in Castration-Resistant State?

When zoledronic acid was approved in 2002, its application was often extrapolated to all states of PCa with osseous metastases. The fact that only patients with hormone-refractory state were included in the registration trial was not always taken into account in daily clinical practice. In the Cancer and Leukemia Group B study (CALGB) 90202, Smith and colleagues prospectively investigated the safety and efficacy of zoledronic acid vs. placebo in a randomized controlled trial in patients with metastatic castration-sensitive PCa [31]. Primary endpoint was time to SRE, defined as radiation to bone, clinical fracture, spinal cord compression, surgery to the bone, or death as a result of PCa. Total accrual was 680 patients. Contrary to castration-resistant state, early zoledronic acid was not associated with an increased time to first SRE. The median time to first SRE was 31.9 months for patients treated with zoledronic acid, compared to 29.8 months in the placebo group (*p* = 0.39). Interestingly, the subgroup analysis for time to SRE showed favourable data for the zoledronic acid group compared to the placebo group in patients with prior SRE (31.9 months vs. 17.6 months; *p* = 0.054). There was no difference in overall survival between both groups (37.9 months vs. 36.0 months; *p* = 0.29). Overall grade 3 or higher treatment-related adverse events were approximately 14% in the zoledronic acid group and 12% in the placebo group.

Regarding denosumab, no efficacy and safety data is available for patients with hormone-sensitive PCa and osseous metastases.

## 8. Treatment Schedules for Bone Protective Agents: Monthly or Three-Monthly?

While treatment recommendation for zoledronic acid is once a year for the prevention of osteoporosis in PCa patients receiving androgen deprivation, the application rate is increased to once per month in CRPC patients with osseous metastases for the prevention of SRE. For denosumab, total dosage per year also increases 12-fold (60 mg twice a year for the prevention of osteoporosis vs. 120 mg monthly for the prevention of SRE in mCRPC). Interestingly, no pharmacokinetic data has been published that clearly shows the need for a 12-fold intensified bone-protective treatment in the presence of osseous metastases. Himelstein and colleagues presented a prospective randomized trial in 1822 cancer patients (breast = 833, prostate = 674, myeloma = 270, and other *n* = 45) testing zoledronic acid monthly vs. 3-monthly for non-inferiority in SRE-prevention [32]. There was no significant difference in the incidence of SRE between monthly and 3-monthly application (29.5% vs. 28.6%; *p* = 0.79). Moreover, dose delays were more common in the monthly-application group (62% vs. 37%). Interestingly, the development of MRONJ was higher in the monthly-application group compared to the 3-monthly-application group (2% vs. 1%). On the other hand, SRE with the indication for bone surgery was higher in patients receiving zoledronic acid every 3 months compared to monthly application (4.6% vs. 2.4%). Although rare, renal deterioration was more present in the monthly application group (1.2% vs. 0.5%). In summary, data is promising to expand the application interval of zoledronic acid from monthly to 3-monthly in patients with mCRPC. Before changing current clinical practice, the full publication of this promising prospective study should be awaited.

## 9. Conclusions

Osseous metastases in patients with PCa are a significant health and economic burden. Every second patient with osseous metastases will develop skeletal-related events, which are associated with an up to 3-fold increased risk for death and doubled treatment costs. Zoledronic acid and denosumab are two potent drugs with the ability to reduce the risk of SRE in CRPC patients with osseous metastases by 11%–16%. However, both drugs do not significantly improve time to tumour progression or overall survival. Both drugs bear the risk of MRONJ, which is approximately 5%. Routine inspections of the oral cavity before, during, and even after treatment with bone protective agents are mandatory to reduce the risk of MRONJ. If dental surgery is indicated, treatment should ideally be completed as long as possible before the application of bone protective agents.

Evaluation of pathological tracer uptake in the jaws on bone scintigraphy routinely performed to assess antitumoral treatment response in CRPC patients improves the detection of early stage MRONJ. In the presence of MRONJ, there is no clear recommendation if bone protective agents have to be stopped or treatment can be proceeded. Zoledronic acid and denosumab are approved for the prevention of SRE in CRPC patients with osseous metastases. Early zoledronic acid in hormone-sensitive state does not significantly delay time to SRE. Early zoledronic acid may play a role in patients with prior SRE. While zoledronic acid and denosumab treatment is usually applied monthly in CRPC patients with osseous metastases, first data shows an extension to 3 months is equi-effective.
